# Finger Millet Ethanol Extracts Prevent Hypertension by Inhibiting the Angiotensin-Converting Enzyme Level and Enhancing the Antioxidant Capacity in Spontaneously Hypertensive Rats

**DOI:** 10.3390/antiox10111766

**Published:** 2021-11-04

**Authors:** Se Yeong Park, Eun Woo Jeong, Yun Sun Yang, Hyun-Joo Kim, Gwang-woong Go, Hyeon Gyu Lee

**Affiliations:** 1 Department of Food and Nutrition, Hanyang University, Seoul 04763, Korea; psydkwk@hanyang.ac.kr (S.Y.P.); bravoadria@hanyang.ac.kr (E.W.J.); diddbstjs777@hanyang.ac.kr (Y.S.Y.); 2 Department of Central Area Crop Science, National Institute of Crop Science, Wanju-Gun 55365, Korea; tlrtod@korea.kr; 3 Korean Living Science Research Center, Hanyang University, Seoul 04763, Korea

**Keywords:** angiotensin-converting enzyme (ACE), antioxidant, hypertension, finger millet, renin

## Abstract

Finger millet (*Eleusine coracana*) contains high levels of calcium and polyphenols, which have a variety of beneficial functions. We tested the hypothesis that finger millet ethanol extracts (FEs) have an antihypertensive effect in spontaneously hypertensive rats (SHRs). The study groups were assigned as follows: (1) Wistar Kyoto rats (normal); (2) SHRs treated with saline (negative control); (3) SHRs treated with captopril 50 mg/kg bw (positive control); (4) SHRs treated with FE 250 mg/kg bw (FE250); and (5) SHRs treated with FE 500 mg/kg bw (FE500). FE supplementation improved the lipid profiles, including the triglyceride, total cholesterol, and low-density lipoprotein cholesterol levels, without deterioration in liver function. The thiobarbituric acid reactive substance concentration and superoxide dismutase activity significantly improved after the application of FE250 and FE500. Interestingly, FE250 and FE500 application dramatically reduced the systolic blood pressure. FE supplementation exhibited powerful control over the renin-angiotensin system by reducing the angiotensin-converting enzyme levels and renin mRNA expression in the kidney. Additionally, FE500 application ameliorated vascular remodeling, reversed the thickening media, and decreased the media thickness/lumen diameter ratio of the aorta. These results imply that FEs are a potent antihypertensive nutraceutical for regulating the renin–angiotensin system and simultaneously inhibiting oxidative stress.

## 1. Introduction

Hypertension is a major cause of disease worldwide [1]. As the blood pressure remains high, the vascular tone increases, and vasoconstriction dysfunction occurs, leading to the need for revascularization [2,3]. The principal causes of hypertension are largely unknown in 80% of patients; this condition is known as essential hypertension [4]. Active research has been conducted to elucidate the association between the blood pressure and oxidative stress. Reactive oxygen species (ROS) production increases in patients with primary hypertension, and excessive ROS induces severe oxidative stress in the kidney and aorta [5]. Moreover, some previous studies have demonstrated a reduction in the blood pressure owing to changes in antioxidant capacity. For instance, antioxidants decrease the blood pressure remarkably by improving the impaired endothelium-dependent relaxation in spontaneously hypertensive rats (SHRs) [6]. Taken together, the antihypertensive effect can be promoted through the mechanism by which natural antioxidants restrain free radicals and ROS.

Angiotensin-converting enzyme (ACE) is a major target for hypertension in the renin–angiotensin-aldosterone system. It converts angiotensin I into angiotensin II, which causes vasoconstriction and blood pressure elevation [7]. For this reason, synthetic ACE inhibitors, such as captopril and enalapril, are mainly used to treat hypertension. However, ACE inhibitors have various side effects, including coughs and skin rashes [8]. Accordingly, interest in finding natural nutraceuticals with ACE-inhibitory activities has recently increased.

Finger millet (*Eleusine coracana*) is a nutritious crop because of its high calcium and polyphenol contents [9]. Previous studies discovered that finger millet seed ethanol extract was rich in phytochemicals such as phenols, tannins, and alkaloids [10]. Moreover, the high antioxidant properties of finger millet ethanol extract were confirmed by scavenging DPPH and ABTS cation radicals [11]. Moreover, it has been reported to have various health benefits, including antitumorigenic, atherosclerogenic, and antimicrobial activities [12,13]. For instance, finger millet improved the glucose levels and antioxidant capacity in a diabetic rat model [14]. Similarly, finger millet protected rats from hyperglycemic and oxidative stresses [15]. As such, the beneficial function of finger millet is explained, along with its high antioxidant capacity.

Nonetheless, the antihypertensive effects of finger millet have not been studied in vivo. Therefore, we hypothesized that finger millet ethanol extracts (FEs) would have the potential to regulate hypertension. The underlying mechanism of FEs is the regulation of ACE level in the renin–angiotensin system by increasing the systemic antioxidant capacity. We used SHRs to test this hypothesis. We examined the growth performance, blood biochemistry assay findings, thiobarbituric acid reactive substance (TBARS) concentration, superoxide dismutase (SOD) activity, and vascular remodeling and explored the molecular phenotype by measuring the mRNA expression of renin in the kidney.

## 2. Materials and Methods

### 2.1. Diets and Animals

Finger millet (*Eleusine coracana*, Finger 1-ho) was obtained from the National Institute of Crop Science (Rural Development Administration, Suwon, Korea). It was ground into a powder form using a blender. The finger millet powder was then mixed with 100% ethanol and stirred overnight at room temperature. The extract was filtered using a filter paper (Advantec No. 2, Advantec Toyo Kaisha Ltd., Tokyo, Japan), concentrated using a rotary evaporator (Eyela NE-2001, Tokyo Rikakikai Co., Tokyo, Japan) below 50 °C, and stored at −80 °C for further experiments.

Six-week-old male Wistar Kyoto (WKY) rats and SHRs were purchased from Central Lab Animal Inc. (Seoul, Korea). All experimental animals were housed at 24 ± 2 °C and a relative humidity of 50 ± 20% with a 12-h light/dark cycle. For the experimental period, the rats were fed a standard diet (SAFE A40, SAFE Inc., Augy, France) and water ad libitum. All experimental procedures were approved by the Institutional Animal Care and Use Committee of the Center at Woojung Bio (Suwon, Korea) under the approval number WJIACUC20200326-1-41.

After one week of acclimation, the rats were assigned to the five groups (*n* = 6): (1) WKY rats treated with saline (WKY, normal control); (2) SHRs treated with saline (SHR, negative control); (3) SHRs treated with 50 mg/kg bw of captopril (Captopril, positive control); (4) SHRs treated with 250 mg/kg bw of FE (FE250); or (5) SHRs treated with 500 mg/kg bw of FE (FE500). Antihypertensive studies in animals administered with FEs have not yet been conducted. Thus, the experimental dosage was selected by citing a similar previous study in which 250 and 500 mg/kg of pearl millet extracts were administered to rats to evaluate the blood-pressure-lowering effect [16]. All experimental animals received their respective treatments via oral administration of samples dissolved with 0.5% carboxymethyl cellulose (Sigma-Aldrich, St. Louis, MO, USA) for 8 weeks. After a 12 h fast, rats were anesthetized using 2% isoflurane (2 L/min). Rats were sacrificed and the kidneys and aorta were harvested immediately. These samples were stored at −80 °C until analysis. 

### 2.2. Growth Performance Analysis

Body weight and food intake were measured weekly. Weight gain was calculated by subtracting the initial weight from the final weight.

### 2.3. Blood Pressure Measurement

Once a week, the systolic (SBP) and diastolic blood pressures (DBP) were recorded by noninvasive tail-cuff method using LE 5002 (Panlab Inc., Barcelona, Spain). A tail-cuff sensor, attached to an amplifier, was connected to the tail. The blood pressure measurement was carried out three times.

### 2.4. Blood Biochemistry Assay

Blood was collected from the caudal vena cava to obtain serum and plasma. The serum levels of aspartate aminotransferase (AST) and alanine transaminase (ALT) were determined using a kits (Asan Pharmaceutical, Seoul, Korea). In addition, the profiles of plasma triglyceride (TG), total cholesterol (TC), and low-density lipoprotein cholesterol (LDL-C) levels were analyzed using an autoanalyzer (Hitachi, Tokyo, Japan). The ACE level (Cusabio Corp., Wuhan, China) in the serum was measured using a rat ELISA kit.

The plasma malondialdehyde was examined using the TBARS assay. First, the plasma was mixed with 10% trichloroacetic acid, and the mixture was placed in an ice bath for 10 min to precipitate the plasma components. Next, the samples were centrifuged at 2200× *g* for 15 min before adding thiobarbituric acid. After boiling the mixture at 100 °C for 10 min, spectrophotometric measurements were recorded at a 532 nm wavelength. The SOD activity in the serum was recorded using the SOD Assay Kit-WST (Dojindo Laboratories, Kumamoto, Japan) according to the manufacturer’s instructions.

### 2.5. Histological Analysis of the Aorta

The rats’ isolated aortic tissues were fixed with 4% paraformaldehyde for 24 h. They were then dehydrated and embedded in paraffin. Next, the aorta was stained with hematoxylin and eosin to determine the media thickness (MT) and lumen diameter (LD) of the aorta. The samples were viewed under a microscope at 20× magnification. The MT and LD of the aorta were determined using the ImageJ software (National Institutes of Health, Bethesda, MD, USA). MT/LD ratio was then calculated based on MT and LD.

### 2.6. Real-Time PCR

Total mRNA was isolated from the kidney with TRIzol (Ambion, Austin, TX, USA) and PureLink RNA Mini Kit (Invitrogen, Carlsbad, CA, USA) according to the manufacturer’s protocol. The concentrations of RNA were determined using a NanoDrop (Thermo Fisher Scientific, Waltham, MA, USA). The purified RNA of each sample was subjected to cDNA synthesis using Prime Script™ RT reagent kit (Takara, Shiga, Japan). PCR was performed with SYBR green to measure the renin mRNA expression using the CFX96TM RT-PCR detection system (Bio-Rad, Hercules, CA, USA). The sequences of primer were as follows: renin (NM_012642.4) forward primer, 5′-TGCTAAAGGAGGAAGTGTTT-3′; renin reverse primer, 5′-TGATGCTCACGTAGTGAAAG-3′; GAPDH (NM_017008.4) forward primer, 5′-GTCGGTGTGAACGGATTTG-3′; GAPDH reverse primer, 5′-TCCCATTCTCAGCCTTGAC-3′. The relative gene expression of renin was quantified using GAPDH. The data were analyzed using the Bio-Rad CFX-Manager software 3.1 (Bio-Rad, Hercules, CA, USA).

### 2.7. Statistical Analysis

All data were expressed as means ± standard errors of the mean (SEM). Data were analyzed using one-way analysis of variance followed by Duncan’s multiple range test (SPSS version 24.0, SPSS Inc., Chicago, IL, USA). Significant differences in the mean values were defined at *p* < 0.05. Graphs were created using GraphPad Prism version 8 (GraphPad Software, La Jolla, CA, USA).

## 3. Results and Discussion

### 3.1. Effects of FE Supplementation on the Growth Performance

The animal model used in this study was an SHR model, which is frequently used in essential hypertension research. The SHR strain was produced by selective inbreeding of WKY rats with a high blood pressure [17]. The growth performances of the rats are shown in Table 1. The body weight and weight gain of the SHRs were significantly lower than those of the WKY rats, despite higher food intake. These observations are consistent with those of a previous study in which the WKY group showed greater weight than the SHR group. Similarly, the feed efficiency rate was lower in the SHRs than in the WKY rats [18]. Thus, these phenomena have been demonstrated to consume more energy because of generic differences [19,20].

Given the successful establishment of an in vivo model, we assessed the effect of FE supplementation on growth performance. The body weights of the FE250 and FE500 groups were not different from those of the SHR group, despite greater food intake. Similarly, there was no significant difference in the body weight of the SHR model classified by legumes or cereals [21,22]. Furthermore, Captopril, a positive control treatment for hypertension, did not alter the body weight or food intake.

### 3.2. Effect of FE Supplementation on the Blood Biochemical Parameters

To investigate the effect of FE supplementation on liver function, we measured the AST and ALT levels in the serum (Table 2). AST and ALT are enzymes that synthesize amino acids in vivo and are mainly present in the liver [23]. They are secreted into the blood when the liver is damaged and are used for liver toxicity research [24]. Accordingly, the activities of AST and ALT were considered to be indicators of the toxicity of the FEs. Herein, the AST and ALT levels were significantly higher in the SHRs than in the WKY rats. In general, elevated levels of liver enzymes are often observed with an increased blood pressure. Previous studies have found that the serum AST and ALT levels were notably higher in the hypertensive group than in the normotensive group [25]. Similarly, the AST and ALT levels in the SHR group increased over time [26]. In contrast, the ALT and AST levels in the FE250 and FE500 groups were within normal ranges (<100 IU/L) [27,28]. Furthermore, the AST level in the FE250 and FE500 groups was similar to that of the WKY group. In sum, these results showed that the administration of FEs had no adverse effects on the liver damage, and the liver damage in the SHRs recovered.

The blood lipid profiles, including the TC, TG, and LDL-C levels, are presented in Table 2. The levels of TG and TC in the SHR group notably increased compared to the WKY group. These results are consistent with the research findings that the TG and TC levels were higher in SHRs than in WKY rats [29,30]. In contrast, the TG and TC levels in the FE250 and FE500 groups notably decreased compared with those in the SHR group. Moreover, the LDL-C level decreased in both the FE250 and FE500 groups compared with that in the SHR group. In particular, the TG and LDL-C levels in the FE500 group were dramatically lower than the SHR group. It was previously reported that reductions in the TG and TC levels in hypertensive rats were associated with decreased SBP and alleviation of atherosclerosis [31]. In addition, the LDL-C level was a major risk factor for atherogenesis and cardiovascular disease in humans, and lowering the LDL-C levels was considered an important treatment [32]. Taken together, FE supplementation has the potential to improve lipid profiles related to the onset of arteriosclerosis.

### 3.3. Effects of FE Supplementation on the Antioxidant Capacity, including the TBARS Concentration and SOD Activity

**Figure 1:**
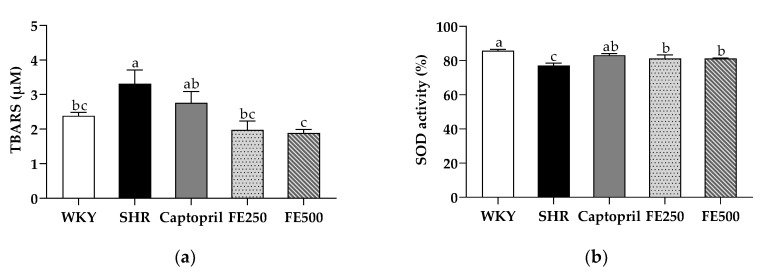
TBARS concentration in the plasma (**a**) and SOD activity in the serum (**b**) in the SHRs after 8 weeks of oral administration. TBARS, thiobarbituric acid reactive substance; SOD, superoxide dismutase; WKY, Wistar Kyoto rats; SHR, Spontaneously hypertensive rats; Captopril, SHRs treated with 50 mg/kg bw of captopril; FE250, SHRs treated with 250 mg/kg bw of FE; FE500, SHRs treated with 500 mg/kg bw of FE; FE, finger millet ethanol extract. Data are expressed as means ± standard errors of the mean (*n* = 6). Different letters above the bars indicate significant differences at *p* < 0.05.

Increased oxidative stress is one of the key provisions of hypertension, which amplifies blood pressure elevation in the presence of the renin-angiotensin system. To investigate the effect of FE supplementation on oxidative stress, we assessed the TBARS concentration and SOD activity in the blood (Figure 1). Previous studies have suggested that the production of ROS and the deficiency of the antioxidant system may contribute to the vascular damage of spontaneously hypertensive rats [6]. Moreover, several studies have demonstrated an association between hypertension and oxidative stress by identifying high ROS levels in animal models of hypertension [33,34]. In addition, an increase in lipid peroxidation according to oxidative stress was confirmed in hypertensive patients [35]. The TBARS concentration in the plasma of the SHR group was significantly increased compared with that of the WKY group. These results indicate that oxidative stress is higher in SHRs than in WKY rats, as previously reported [22,36]. Both FE groups showed a significantly decreased TBARS concentration in the plasma compared with the SHR group. In particular, the FE500 group exhibited a robust lipid peroxidation inhibitory activity (57%). In previous studies, finger millet contained a sufficient amount of antioxidant compounds [37,38]. Moreover, the antioxidant effect of a millet-enriched diet reduced the blood pressure in rats [39]. These results show that FEs are a rich antioxidant source for reducing the TBARS concentration in SHRs.

ROS generation influenced the blood vessels, kidney, and heart in patients with hypertension, increasing the blood pressure and cardiac output [40]. Previous studies have shown that SOD had an oxidative effect on ROS by converting ROS into hydrogen peroxide and oxygen [41]. In our study, the SOD activity in the SHR group decreased compared with that in the WKY group, as expected. However, the SOD activity in the serum was strikingly upregulated in the FE250 and FE500 groups rather than in the SHR group. Similarly, hypertension was previously rescued by acacia polyphenol in SHRs to enhance the SOD activity [42]. In summary, our results indicate that the antioxidant potential of FEs promotes their antihypertensive effect.

### 3.4. Effects of FE Supplementation on the Blood Pressure

**Figure 2:**
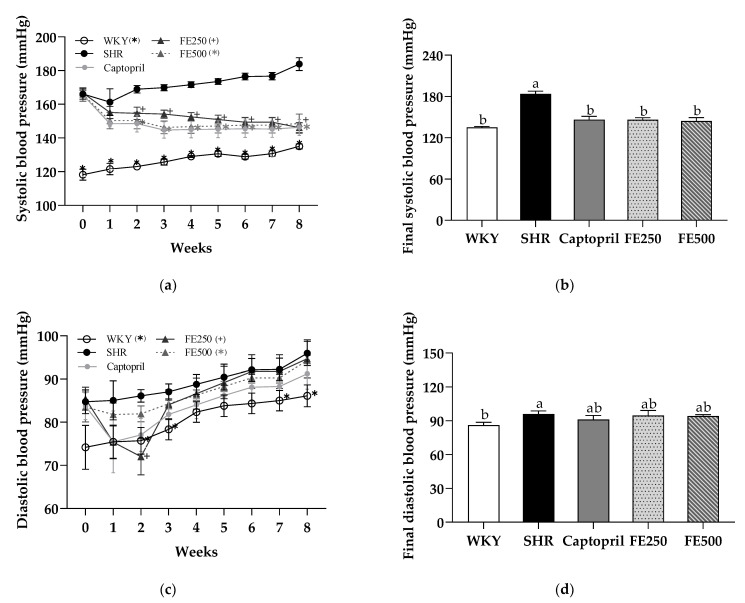
Systolic blood pressure (**a**), final systolic blood pressure (**b**), diastolic blood pressure (**c**) and final diastolic blood pressure (**d**) in the SHRs during 8 weeks of oral administration. WKY, Wistar Kyoto rats; SHR, Spontaneously hypertensive rats; Captopril, SHRs treated with 50 mg/kg bw of captopril; FE250, SHRs treated with 250 mg/kg bw of FE; FE500, SHRs treated with 500 mg/kg bw of FE; FE, finger millet ethanol extract. Data are expressed as means ± standard errors of the mean (*n* = 6). Different letters above the bars indicate significant differences at *p* < 0.05.

The effect of FE supplementation for 8 weeks on the SBP and DBP was examined (Figure 2). The SBP and DBP are important overall predictors of cardiovascular risk in patients with hypertension. Therefore, the SHR model is a suitable model for studying essential hypertension [43]. The SBP and DBP in the SHR group were higher than those in the WKY group, indicating the successful establishment of the hypertensive model (Figure 2a,c). Furthermore, the SBP in the Captopril, FE250, and FE500 groups dramatically decreased compared with that in the SHR group at week 2. Surprisingly, at the end of the experiment, the SBP in the FE250 (20%) and FE500 groups (21%) reduced to a level similar to that in the Captopril group (Figure 2b). This finding was first reported herein; previously, similar varieties of foxtail millet (*Setaria italica*) reduced the blood pressure in subjects with mild hypertension. However, the DBP did not change after FE supplementation (Figure 2d). In conclusion, the SBP-lowering effect of FE supplementation showed that finger millet could be a potential functional food for improving the blood pressure.

### 3.5. Effects of FE Supplementation on the ACE Level and Renin Activity

**Figure 3:**
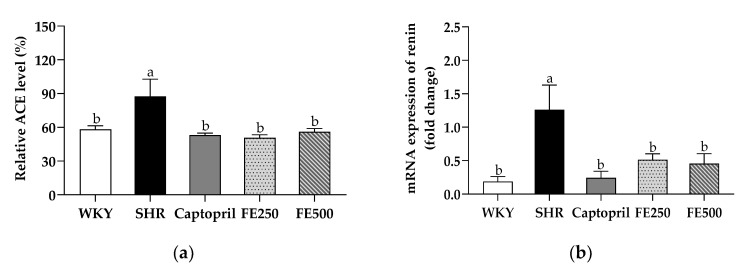
ACE level in the serum (**a**) and mRNA expression of renin in the kidney (**b**) of the SHRs after 8 weeks of oral administration. ACE, angiotensin-converting enzyme; WKY, Wistar Kyoto rats; SHR, Spontaneously hypertensive rats; Captopril, SHRs treated with 50 mg/kg bw of captopril; FE250, SHRs treated with 250 mg/kg bw of FE; FE500, SHRs treated with 500 mg/kg bw of FE; FE, finger millet ethanol extract. Data are expressed as means ± standard errors of the mean (*n* = 6). Different letters above the bars indicate significant differences at *p* < 0.05.

To further investigate the effects of FE supplementation on the blood pressure, we evaluated the activity of ACE, which is regarded as the most important enzyme in the renin–angiotensin system (Figure 3a). As predicted, the SHR group showed higher ACE levels than the WKY group. These observations are consistent with those of previous studies showing that the ACE activity was higher in SHRs than in WKY rats [18,44]. Meanwhile, the Captopril, FE250, and FE500 groups showed significantly lower ACE levels than the SHR group (53%, 50%, and 56%, respectively); the FE250 and FE500 groups showed a similar inhibition of the ACE level as the Captopril group. Further studies evaluating the ACE level in the tissues, including the lung, kidney, and aorta, are needed to clarify how FE supplementation lowers the blood pressure. In conclusion, FE supplementation remarkably reduced the ACE level, which was considered the main element of the blood pressure rescue presented earlier.

To investigate the molecular evidence of the effect of FE supplementation in the renin–angiotensin system, we assessed the mRNA expression of renin in the kidney (Figure 3b). The kidney is a pivotal organ for blood pressure regulation [45]. The primary function of renin is to increase the blood pressure by restoring the perfusion pressure in the kidney. Accordingly, a reduction in the blood pressure is intimately associated with renin inhibition [46]. The renin mRNA expression in the kidney of the SHR group was enhanced compared with that of the WKY group, which is consistent with previous findings [47]. Conversely, the renin mRNA expression in the kidney was significantly suppressed in the FE250 and FE500 groups compared with that in the SHR group. These results suggest that FE supplementation increased renal perfusion, followed by inhibiting renin expression, intimating reduced blood pressure.

### 3.6. Effect of FE Supplementation on Vascular Remodeling in the SHRs

**Figure 4:**
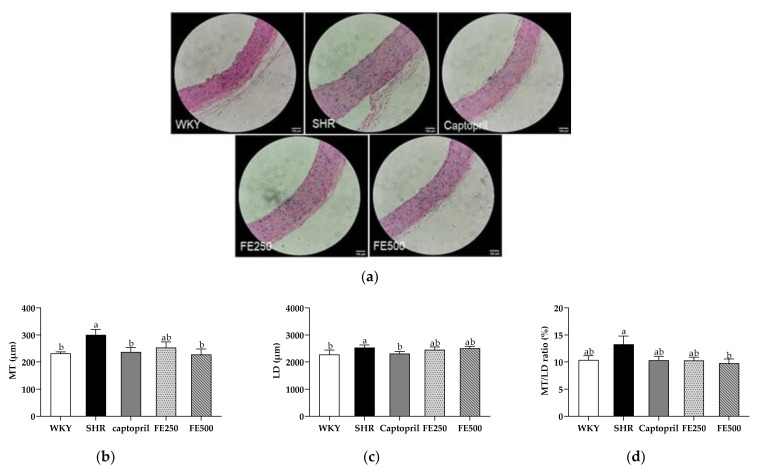
Effect of FE supplementation on aortic remodeling. (**a**) Histopathological changes in the aorta in each group were observed via hematoxylin and eosin staining; (**b**) The MT, (**c**) LD, and (**d**) MT/LD ratio were measured. MT, media thickness; LD, lumen diameter; WKY, Wistar Kyoto rats; SHR, Spontaneously hypertensive rats; Captopril, SHRs treated with 50 mg/kg bw of captopril; FE250, SHRs treated with 250 mg/kg bw of FE; FE500, SHRs treated with 500 mg/kg bw of FE; FE, finger millet ethanol extract. Data are expressed as means ± standard errors of the mean (*n* = 6). Different letters above the bars indicate significant differences at *p* < 0.05.

As blood pressure reduction and ACE activity inhibition were validated, vascular remodeling was examined. The aorta was stained with hematoxylin and eosin to determine the histological changes in the aorta. The MT, LD, and MT/LD ratio were analyzed (Figure 4). An increased MT of the aorta is a common structural feature of hypertensive resistance vessels [48]. Herein, the MT of the aorta in the SHR group was significantly increased compared with that in the WKY group, indicating vascular remodeling in the SHRs. Meanwhile, the Captopril group had a reduced aortic MT compared with the SHR group. Based on previous findings, captopril improves the blood pressure by thinning the aortic wall [49]. Interestingly, the MT of the aorta in the FE500 group was significantly lower than that in the SHR group. There was no significant difference in the LD between the FE-supplemented groups. Previous studies have shown that the MT/LD ratio in SHRs is higher than that in WKY rats [50]. However, the significant reduction in the MT/LD ratio between the SHR and FE500 groups in our study indicated improved vascular remodeling. There have been no studies on the effect of finger millet supplementation on vascular remodeling caused by hypertension. However, a previous study has found that major polyphenolic components, such as 3-hydroxybenzylhydrazine, luteolin-3′,7-diglucoside, p-coumaric acid, and other polyphenols in millet shells, inhibit the development of atherosclerotic plaques in the aorta. Another study reported that peroxidase derived from foxtail millet bran improved atherosclerosis by inhibiting aortic sinus lesions in ApoE−/− mice [51]. In conclusion, FE supplementation ameliorates vascular remodeling in SHRs, including the MT and MT/LD ratio of the aorta, suggesting that it could modulate the blood pressure by improving vascular remodeling.

## 4. Conclusions

FE supplementation dramatically diminished the SBP of the SHRs. It exhibited powerful control over the renin–angiotensin system by reducing the ACE level and renin mRNA expression in the kidney. In addition, FE500 application ameliorated vascular remodeling, reversed the thickening media, and decreased the MT/LD ratio of the aorta. We also confirmed the antioxidant effects of FEs, which improved the TBARS concentration and SOD activity. In conclusion, FEs are a new functional ingredient for lowering high blood pressures by regulating the renin–angiotensin system and simultaneously inhibiting oxidative stress. We may conduct further studies to identify specific phenolic compounds with antihypertensive effects before their actual application in hypertensive patients. In addition, more studies using mechanism analysis are needed to further understand the correlation between antioxidant activity and antihypertensive effects.

## Figures and Tables

**Table 1:** Effects of FE supplementation on the growth performance of the WKYs and SHRs.

	WKY	SHR	Captopril	FE250	FE500
Final body weight (g)	377.1 ± 2.73 ^a^	344.7 ± 6.09 ^b^	350.5 ± 6.10 ^b^	352.9 ± 8.52 ^b^	346.7 ± 3.78 ^b^
Weight gain (g)	17.58 ± 0.53 ^a^	12.78 ± 0.18 ^c^	14.05 ± 0.70 ^bc^	15.50 ± 0.70 ^b^	15.48 ± 0.52 ^b^
Food intake (g/week)	74.19 ± 0.74 ^c^	79.21 ± 0.92 ^b^	79.42 ± 1.05 ^b^	82.91 ± 0.66 ^a^	81.84 ± 0.41 ^a^

WKY, Wistar Kyoto rats; SHR, Spontaneously hypertensive rats; Captopril, SHRs treated with 50 mg/kg bw of captopril; FE250, SHRs treated with 250 mg/kg bw of FE; FE500, SHRs treated with 500 mg/kg bw of FE; FE, finger millet ethanol extract. Data are expressed as means ± standard errors of the mean (*n* = 6). ^a–c^ The values with different superscript letters within a row are significantly different at *p* < 0.05.

**Table 2:** Effects of FE supplementation on the serum and plasma biochemical parameters in the WKYs and SHRs.

	WKY	SHR	Captopril	FE250	FE500
AST level (IU/L)	18.57 ± 1.87 ^b^	24.71 ± 1.94 ^a^	17.97 ± 1.57 ^b^	15.94 ± 2.65 ^b^	13.85 ± 1.08 ^b^
ALT level (IU/L)	4.285 ± 0.76	33.27 ± 14.1	8.045 ± 1.16	19.59 ± 6.45	14.95 ± 2.41
TG level (mg/dL)	42.51 ± 5.91 ^c^	135.0 ± 7.24 ^a^	74.46 ± 6.85 ^b^	91.98 ± 18.1 ^b^	83.41 ± 2.54 ^b^
TC level (mg/dL)	88.36 ± 2.18 ^b^	95.55 ± 1.20 ^a^	77.74 ± 0.88 ^d^	80.68 ± 1.29 ^c,d^	83.41 ± 2.54 ^b,c^
LDL-C level (mg/dL)	8.95 ± 0.25 ^b^	9.84 ± 0.38 ^a^	7.73 ± 0.15 ^c^	8.35 ± 0.39 ^b,c^	7.54 ± 0.11 ^c^

WKY, Wistar Kyoto rats; SHR, Spontaneously hypertensive rats; Captopril, SHRs treated with 50 mg/kg bw of captopril; FE250, SHRs treated with 250 mg/kg bw of FE; FE500, SHRs treated with 500 mg/kg bw of FE; FE, finger millet ethanol extract; AST, aspartate transaminase; ALT, alanine aminotransferase; TG, triglyceride; TC, total cholesterol; LDL-C, low-density lipoprotein cholesterol. Data are expressed as means ± standard errors of the mean (*n* = 6). ^a–d^ The values with different superscript letters within a row are significantly different at *p* < 0.05.

## Data Availability

The data presented in this study are available within the article.
